# The Impact of Perspective Taking on Obesity Stereotypes: The Dual Mediating Effects of Self-Other Overlap and Empathy

**DOI:** 10.3389/fpsyg.2021.643708

**Published:** 2021-08-12

**Authors:** Yunlong Wu, Yuzhu Zhang

**Affiliations:** School of Psychology, Inner Mongolia Normal University, Hohhot, China

**Keywords:** obesity stereotypes, perspective taking, empathy, self-other overlap, dual mediating effects

## Abstract

Previous studies have indicated that obese people face many forms of severe prejudice and discrimination in various settings, such as education, employment, and interpersonal relationships. However, research aimed at reducing obesity stereotyping is relatively rare, and prior studies have focused primarily on negative stereotypes. Based on the empathy-altruism hypothesis and self-other overlap hypothesis, this study investigates the impact of perspective taking (PT) on both positive and negative obesity stereotypes and examines the mediating effects of empathy and self-other overlap. A sample of 687 students (191 males and 496 females) at Chinese universities participated by completing self-report questionnaires on trait tendency and evaluation toward obese people. Structural equation modeling and the bootstrap method revealed that self-other overlap (but not empathy) mediated the relationship between PT and negative obesity stereotypes. While self-other overlap and empathy both mediated the relationship between PT and positive obesity stereotypes. These findings address the importance of PT for improving positive and negative obesity stereotypes: specifically, PT promotes psychological merging, and produces empathic concern (EC).

## Introduction

Obese people are severely stigmatized because of their weight. An intensive review by Puhl and Heuer (2009) shows that they confront prejudice and discrimination in many forms. In educational settings, obese students are less likely than normal-weight students to obtain a college degree (Fowler-Brown et al., 2010) or be accepted as graduate students after an interview (Burmeister et al., 2013). As regards interpersonal relationships, obese individuals tend to have poor relationships with their peers in school (Puhl and Latner, 2007) and have fewer close friends relative to thinner individuals (Sarlio-Lähteenkorva, 2001). Further, in employment settings, obese applicants are sometimes not hired because of discrimination based on their weight (Puhl et al., 2008). Individuals experiencing the stigma of obesity are vulnerable to psychological disorders such as depression, low self-esteem, and anxiety (Puhl and King, 2013). This stigma derives partly from negative stereotypes (Tiggemann and Anesbury, 2000) that obese people are lazy, unintelligent, and lacking in self-discipline and willpower (Puhl and Heuer, 2009). Although there is a lot of evidence about the adverse consequences of the stigma of obesity, relatively few studies have explored strategies to reduce obesity stereotypes and prejudices (Gloor and Puhl, 2016). It is, therefore, necessary to take measures to improve attitudes toward this group.

### Perspective Taking (PT) and Stereotypes

Corrigan and O'Shaughnessy (2007) propose three main avenues to change stereotypes and stigma, namely protest, education, and contact. However, the protest may provoke rebound effects, arouse resistance, and ultimately fail to induce more positive attitudes (Corrigan et al., 2001). Furthermore, the magnitude and duration of education to improve attitudes may be restricted (Corrigan et al., 2002). For contact to be effective, some optimal conditions are required, such as shared goals and cooperation (Pettigrew and Tropp, 2006). Different from these aforementioned approaches (e.g., PT will not cause the rebound effects), PT has become an effective stereotype-improvement strategy (Galinsky, 2002; Todd and Galinsky, 2014; Sun et al., 2016; Wang et al., 2018; Huang et al., 2020) because of its unique advantages. Specifically, its positive effect can be generalized from specific individuals to the groups they belong to Shih et al. (2009) for a relatively long time (Batson et al., 1997) at both the explicit and implicit levels (Todd and Burgmer, 2013). The limited existing work applied this strategy to anti-fat bias found that taking the perspective of obese persons who experienced discrimination did produce reduced implicit bias among overweight participants (Teachman et al., 2003). There are two main theoretical hypotheses to explain the mechanisms underlying PT.

### PT, Empathy, and Stereotypes

The first is the empathy-altruism hypothesis (Batson et al., 1997), focused on the emotional level, which holds that taking the perspective of a member of a stigmatized group will increase the empathic feelings of an individual, such as sympathy and compassion for that individual. These feelings will evoke altruistic motivation and generalize to the whole stigmatized group, thus increasing the positive evaluation and attitude of an individual toward the group. The theory that PT indirectly reduces stereotyping through the mediating effect of empathy has been tested in many groups, such as people with AIDS (Li, 2017), drug addicts (Batson et al., 2002), African Americans (Vescio et al., 2003), homosexuals (Wang, 2018), and the elderly (Bian, 2015). However, few studies have applied this method to attempt to tackle obesity stereotyping. As far as the author knows, only Cheng and Zhang (2017) have examined the direct impact of PT on implicit obesity stereotypes in China; they found significant attenuation of stereotyping but did not explore the underlying mechanism. To address this deficiency, the present study draws on the empathy-altruism hypothesis to test the mediating role of empathy between PT and obesity stereotypes. Another limitation in the literature is that many studies have shown the role of PT in reducing negative stereotypes (Galinsky and Moskowitz, 2000; Ku et al., 2010; Todd et al., 2011) but few have considered positive stereotypes (Wang et al., 2014). Obesity stereotypes include not only negative qualities but also positive characteristics, such as warmth and friendliness (Tiggemann and Rothblum, 1988). Does PT weaken or enhance positive obesity stereotypes? The current study will answer this question.

### PT, Self-Other Overlap, and Stereotypes

The second explanation mechanism is the self-other overlap hypothesis (Davis et al., 1996; Galinsky and Moskowitz, 2000), focused on the cognitive level, which holds that PT can activate self-concept and lead perspective takers to attribute a greater proportion of their self-traits to the other. With perspective takers perceiving that they share more common characteristics with the target, they merge their mental representations of self and others, which changes their evaluation of the target group. Galinsky and Ku (2004) indirectly tested the self-other overlap hypothesis by examining the moderating role of self-esteem on the effect of PT on prejudice toward the elderly. They reasoned that because perspective takers change their attitude by applying the self to the target, the positive self will be activated and applied to the target group when the perspective takers have high self-esteem, thereby improving their attitude toward target group members; conversely, when perspective takers feel negative about themselves, there is no reduction in prejudice against the target group. As expected, Galinsky and Ku (2004) found that perspective takers with high self-esteem evaluated the elderly more positively than did those with low self-esteem. However, the studies of Bian (2015), Li (2017), and Wang (2018), did not produce this result. The three researchers suggested that the inconsistent findings may be caused by a cultural difference in self-esteem between East and West. Todd et al. (2012) found that automatic self–Black associations, measured by IAT, mediated the effect of PT on perceptions of racial discrimination. Additionally, there are multiple measures to assess self–other overlap, such as Adjective Checklist Overlap, Absolute Difference in Attribute Ratings, and the Inclusion of Others in Self Scale (IOS; Aron et al., 1992). Myers and Hodges (2012) comprehensively analyzed these measures and identified perceived closeness (e.g., IOS) as the most effective way to measure the quality of the relationship between two people. For the above reasons, the present study does not take the indirect approaches but, instead, uses the IOS to directly measure self-other overlap. The aim is to test whether the influence of PT on obesity stereotypes is realized through the mediating effect of self-other overlap.

In summary, based on the empathy-altruism hypothesis and self-other overlap hypothesis, this study investigates the impact of PT on positive and negative obesity stereotypes and examines the underlying mechanism of this relationship, namely the mediating effects of empathy, and self-other overlap.

## Methods

### Participants

The participants were 687 undergraduates recruited from three universities in Hebei, Shandong, and Jiangxi Province, China, comprising 191 males and 496 females. The average age of participants was 19.91 years (SD = 1.14, age-range = 17~24). Among the participants, 151 students majored in Chinese language and literature, 378 students majored in pharmacy, and 158 students majored in education. Six hundred forty eight students were Han and the rest were ethnic minorities. In terms of self-reported body weight, 141 participants were underweight, 389 participants were moderate, and 157 participants were overweight.

### Procedure

A teacher from each University conducted the questionnaire survey in class. The teachers first explained to the students their rights as participants, including the option to withdraw and assured them that their anonymity and the confidentiality of their responses were guaranteed. Written informed consent was obtained from all participants; for underage participants, consent was obtained from parents. Teachers then distributed the questionnaire to participants, who were seated separately and not permitted to interact with one another while completing the questionnaire. Each participant received a small reward for their contribution to the study.

### Measures

#### PT and Empathy

Following Wang et al. (2014), this study used the PT and empathic concern (EC) subscales of the Chinese version of Zhan (1986) of the Interpersonal Reactivity Index (Davis, 1980) to respectively measure the PT and empathy tendency of students. The PT subscale contains five items, such as “I sometimes try to understand my friends better by imagining how things look from their perspective”; the EC subscale includes six items, such as “I often have tender, concerned feelings for people less fortunate than me.” All items are measured using a 5-point Likert scale of agreement, with response options ranging from 0 (“does not describe me well”) to 4 (“describes me very well”). Cronbach's alphas for each subscale were α_PT_ = 0.84, α_EC_ = 0.52, consistent with the previous study (e.g., Zhang et al., 2010).

#### Self-Other Overlap

Self-other overlap was measured by the IOS. The scale comprises seven pairs of circles with an increasing degree of overlap. One circle represents the self and the other represents obese group members. Participants were asked to select which pair of circles best described their relationship with the obesity group. The scale is scored from 1 (no overlap) to 7 (nearly complete overlap). The higher the overlapping degree of circles, the greater the self-other overlap.

#### Obesity Stereotypes

Obesity stereotypes were selected from the studies of Vartanian et al. (2015) and Cheng (2017). Specifically, this study included five negative stereotypes (lazy, sloppy, self-indulgent, lacking in self-discipline, and clumsy) and five positive stereotypes (kind, warm, optimistic, simple and honest, and generous). Participants were asked to rate the extent to which each stereotype fitted the characteristics of obese people. The response options ranged from 1 = strongly disagree to 5 = strongly agree. Cronbach's alphas for the overall scale and each subscale were α_overall_ = 0.77, α_positivestereotypes_ = 0.84, and α_negativestereotypes_ = 0.90.

### Analytic Strategy

Descriptive statistics were performed using SPSS 22. The hypothesized relationships were tested through a series of structural equation models and the bootstrap method in AMOS. Studies using structural equation modeling usually set several thresholds for goodness-of-fit indexes, such as root mean square error of approximation (RMSEA) ≤ 0.08 (Williams et al., 2009), CFI ≥ 0.90, and 2 ≤ χ^2^/df ≤ 5 (Hair et al., 2010). Before the data analysis, all variables were standardized. The items of PT, empathy, self-other overlap, and obesity stereotypes were used as analysis indicators. Self-other overlap was considered as a manifest variable, while PT, empathy, and obesity stereotypes were considered as latent variables.

## Results

### Control for Common Method Bias

Through precautions such as protecting anonymity and using various response formats (5- and 7-point scales), the data collection process was designed to minimize the common method bias of data obtained from the self-report questionnaire. Following data collection, Harman's single-factor test (Podsakoff et al., 2003) was conducted to control for common method bias. The results revealed five factors with eigenvalues >1, of which the first factor accounted for 20.16% of the total variance (below the critical standard of 40%). Therefore, common method bias was not a significant problem in this study.

### Descriptive Statistics

The ranges, means, SDs, and correlations of the research variables are reported in Table 1. All the variables were significantly correlated, except empathy–negative stereotypes and negative–positive stereotypes. In addition, the age and gender of participants were not significantly correlated with either dependent variable [negative (*r*_age_ < −0.01, *p* = 0.95; *r*_gender_ = 0.05, *p* = 0.20] and positive (*r*_age_ = 0.04, *p* = 0.25; *r*_gender_ <0.01, *p* = 0.99) obesity stereotypes; for simplicity, these results are not presented in the Table. The body weight of participants was significantly correlated with the positive obesity stereotypes (*r* = 0.10, *p* < 0.05), so body weight was controlled for in the subsequent analysis.

**Table 1:** Descriptive statistics of the sample.

**Variables**	**Range**	***M (Min–Max)***	***SD***	**1**	**2**	**3**	**4**	**5**
1. Perspective taking	3.80	3.33 (1.20–5.00)	0.79	1				
2. Empathy	3.17	3.83 (1.83–5.00)	0.55	0.30^***^	1			
3. Self-other overlap	6.00	3.85 (1.00–7.00)	1.81	0.25^***^	0.18^***^	1		
4. Negative stereotypes	4.00	2.61 (1.00–5.00)	0.85	−0.13^**^	−0.05	−0.15^***^	1	
5. Positive stereotypes	4.00	3.62 (1.00–5.00)	0.86	0.12^**^	0.11^**^	0.13^**^	−0.008	1

*N = 687*.

*p < 0.05;

** *p < 0.01*;

*** *p < 0.001*.

### Analysis of Mediating Effect

**Figure 1:**
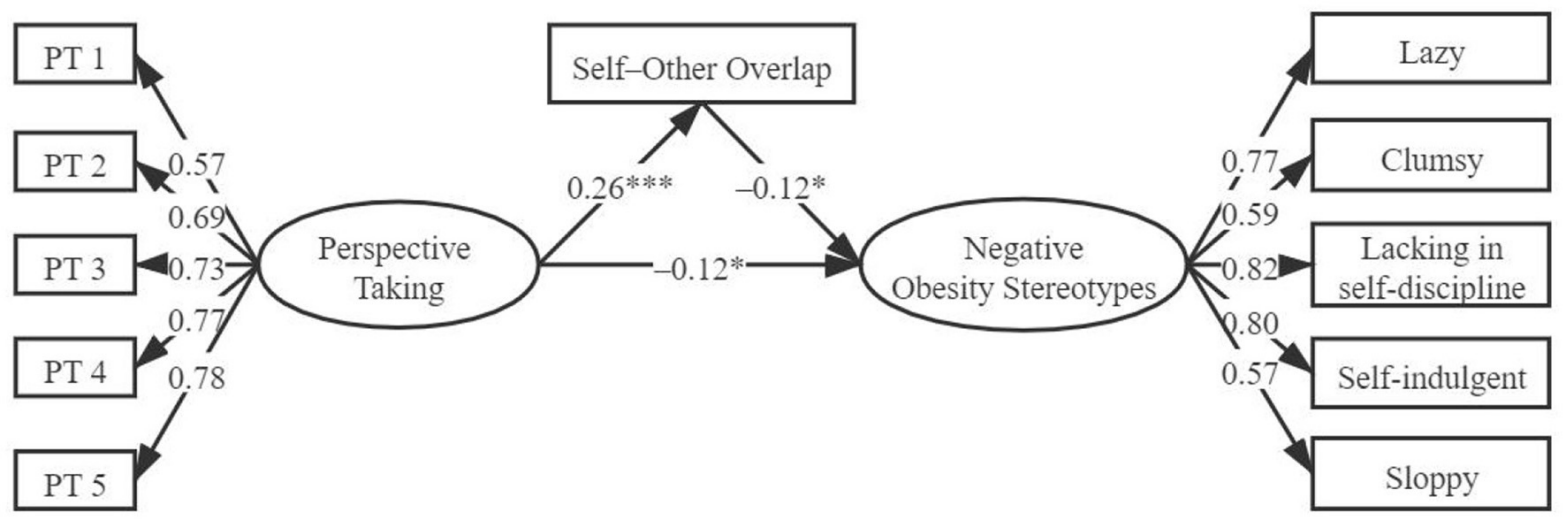
The mediating role of self-other overlap between perspective taking and negative obesity stereotypes. ^*^p < 0.05; ^***^p < 0.001.

First, the influence of PT on the negative obesity stereotypes and the mediating mechanism of this relationship were examined. Step 1 tested the direct effect of PT on negative obesity stereotypes. The results showed that the direct path was significant (β = −0.15, *p* < 0.001) but the model fit was poor (RMSEA = 0.10, χ^2^/*df* = 7.95). Step 2 then assessed the mediating role of empathy between PT and negative obesity stereotypes. As reported above, the correlation between empathy and negative obesity stereotypes was not significant, meaning that PT could not affect the negative obesity stereotypes through the mediating role of empathy. The mediation model analysis also supported this result: Although the indirect path from PT to empathy was significant (β = 0.59, *p* < 0.001), the indirect path from empathy to negative obesity stereotypes did not reach statistical significance (β = 0.001, *p* > 0.05). Next, step 3 examined the mediating role of self-other overlap in the effect of PT on negative obesity stereotypes. Model analysis showed that each path reached a significant level (as shown in Figure 1) and the model fitted well (RMSEA = 0.07, goodness of fit index (GFI) = 0.96, incremental fit index (IFI) = 0.95, comparative fit index (CFI) = 0.95, tucker-lewis index (TLI) = 0.94, chi-square/degree of freedom (the minimum discrepancy divided by degrees of freedom) χ^2^/*df* = 4.23). The mediating effect was tested by the bootstrap method. The results showed that the mediating effect of self-other overlap was significant with an indirect effect of 0.03 and a 95% confidence interval (CI) of [−0.06, −0.01]. Then pwrSEM was used to conduct a power analysis (Wang and Rhemtulla, 2021). Results suggested that the study had 0.81 power to detect an indirect effect of 0.03 in the model. The above results indicate that PT has a negative effect on negative obesity stereotypes through the mediating effect of self-other overlap. However, empathy appears not to play a mediating role.

Second, the influence of PT on positive obesity stereotypes and the mediating mechanism of this relationship were examined. The body weight of participants was treated as a control variable because it was identified earlier as being significantly correlated with positive obesity stereotypes. Step 1 tested the direct effect of PT on positive obesity stereotypes. The results indicated that the direct path was significant (β = 0.15, *p* < 0.001) and the model fitted well (RMSEA = 0.07, GFI = 0.95, IFI = 0.96, CFI = 0.96, TLI = 0.95, χ^2^/*df* = 4.22). Step 2 then assessed the mediating role of empathy between PT and positive obesity stereotypes. The model analysis showed that after adding the mediating variable empathy, the direct path from PT to positive obesity stereotypes became insignificant, while the indirect path from PT to empathy (β = 0.59, *p* < 0.001) and from empathy to positive obesity stereotypes (β = 0.17, *p* = 0.01) reached a significant level. The model fit indexes were RMSEA = 0.07, GFI = 0.92, IFI = 0.91, CFI = 0.91, χ^2^/*df* = 4.49, suggesting that the mediating model acceptably fitted the data. The mediating effect was (0.59 × 0.17)/0.15 = 0.67.

**Figure 2:**
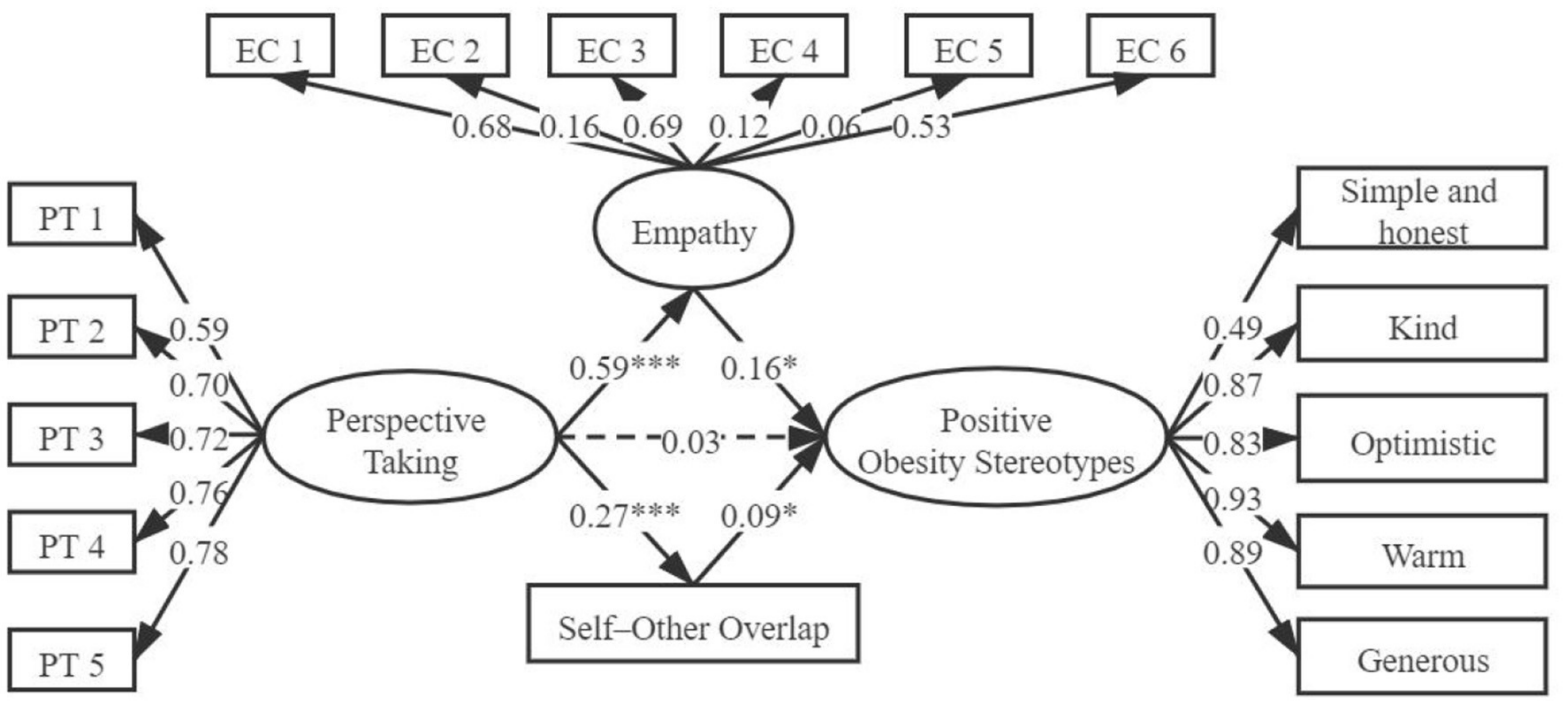
The mediating role of empathy and self-other overlap between perspective taking and positive obesity stereotypes (The dotted line means that the path is not significant). ^*^p < 0.05; ^***^p < 0.001.

Next, step 3 assessed the mediating role of self-other overlap between PT and positive obesity stereotypes. The results showed that after adding the mediating variable self-other overlap, the direct path from PT to positive obesity stereotypes (β = 0.13, *p* < 0.01) and the indirect path from PT to self-other overlap (β = 0.26, *p* < 0.001) and from self-other overlap to positive obesity stereotypes (β = 0.10, *p* < 0.05) were all significant. The measurement model showed acceptable fit: RMSEA = 0.06, GFI = 0.95, IFI = 0.96, CFI = 0.96, χ^2^/*df* = 3.80. The mediating effect was (0.26 × 0.10)/0.15 = 0.17. In step 4, the mediating variables empathy and self-other overlap were combined to construct and test an integrated mediating model. The bootstrap method was used to test multiple mediating effects (Lau and Cheung, 2012). Model analysis indicated that the direct path from PT to positive obesity stereotypes became insignificant when the two mediating variables were added simultaneously. The direct effect was 0.03 and the 95% CI (−0.10, 0.16) contained zero. All the other paths were significant: from PT to empathy [CI: (0.51, 0.67)], from empathy to positive obesity stereotypes [CI: (0.02, 0.31)], from PT to self-other overlap [CI: (0.19, 0.35)], from self-other overlap to positive obesity stereotypes [CI: (0.01, 0.310)]. Finally, a dual mediating model was obtained, with an indirect effect of 0.12, a 95% CI of (0.04, 0.22), and good model fit to the data (as shown in Figure 2, which excludes the control variable for simplicity; RMSEA = 0.07, GFI = 0.92, IFI = 0.91, CFI = 0.91, TLI = 0.90, χ^2^/*df* = 4.16). Then pwrSEM was used to run a power analysis (Wang and Rhemtulla, 2021). Results suggested that the study had 0.85 power to detect an indirect effect of 0.12 in the model. These results suggest that self–other overlap and empathy both play a mediating role between PT and positive obesity stereotypes.

## Discussion

This research aimed to discover the influence of PT on positive and negative obesity stereotypes among University students. Based on the self-other overlap hypothesis (cognitive level) and empathy-altruism hypothesis (emotional level), this study tested the mediating role of self-other overlap and empathy in the relationship between PT and obesity stereotypes. The results will be discussed in detail below.

First, analysis of the mechanism through which PT influences negative obesity stereotypes revealed that self-other overlap, but not empathy, played a mediating role. Although PT can predict empathy, empathy did not significantly affect negative obesity stereotypes. This is consistent with the findings of previous studies that empathy cannot effectively reduce obesity prejudice (Teachman et al., 2003; Gapinski et al., 2006). In this regard, studies suggest that empathy inadvertently emphasizes or evokes the negative aspects of obesity (Daníelsdóttir et al., 2010), which may offset the sympathy and compassion that empathy generates for stigmatized groups in negative evaluations. The mean score of the negative obesity stereotypes in this study was 2.61 (median = 3), indicating that the University students did not consider the negative traits to be characteristic of obese people. If this truly reflects the perception of participants about obese people, then they would feel no need to sympathize. However, the responses of participants may have been affected by social desirability bias, such that even in an anonymous context they did not report their true attitudes with respect to negative obesity stereotypes. Future research will benefit from directly measuring social desirability, to quantify and separate its impact. Another consideration is that this study used a trait tendency measurement, rather than taking a specific perspective for the obese group. This may have prevented participants from putting themselves in the position of obese people to feel the challenges they encounter. Future studies could address this limitation by using experimental methods to manipulate participants into taking the perspective of and empathizing with obese people. It would be valuable to discover whether this approach further verifies the hypotheses and results of this research. Additionally, from the perspective of the common ingroup identity model (Gaertner et al., 1993), recategorizing two separate groups as one group could improve negative evaluations toward outgroup members. By promoting self-other overlapping, the attitudes of perspective takers toward former outgroup members (i.e., obese group) become more positive through processes involving pro-ingroup bias.

Second, this study found that self-other overlap and empathy both mediated the relationship between PT and positive obesity stereotypes. Individuals usually view themselves positively (Taylor and Brown, 1988). Through PT that enhances the psychological merging between self and target, individuals apply the positive description of themselves to obese people, thereby enhancing positive evaluation. This is consistent with the findings of Laurent and Myers (2011) but contrary to the result of Wang et al. (2014), who found that PT reduces the positive stereotypes of doctors through self-other overlap. These different findings may be attributed to the different contents of the stereotype: whereas the traits examined by Laurent and Myers (e.g., attractive, wholesome) and in this study (e.g., kind, warm) mainly focus on the “warmth” dimension, Wang et al. (2014) examined positive characteristics of the “competence” dimension (e.g., analytical, smart).

Although empathy did not mediate the relationship between PT and negative obesity stereotypes, this study supported the empathy-altruism hypothesis by showing that empathy played a mediating role between PT and positive obesity stereotypes. This seems to indicate the positive trait bias of the hypothesis in the obese group. In other words, empathy generates altruistic motivation to help obese people escape unfavorable situations by enlarging their positive traits. However, Gloor and Puhl (2016) found that although PT increases empathy, it also increases fat phobia. This may be related to the attitudes of participants toward obese people. The mean score of the positive obesity stereotypes in this study was 3.62 (median = 3), meaning that participants consider the positive traits to be fairly characteristic of obese people. Skorinko and Sinclair (2013) pointed out that when the characteristics of the target group are consistent with the stereotypes, PT will cause stereotyping to increase.

Finally, the shortcomings of this study must be acknowledged. Obesity stereotypes were measured through a self-report questionnaire. In some prior studies, participants who did not show explicit obesity prejudice were found to have strong implicit negative obesity stereotypes (Teachman et al., 2003). Therefore, future research should use implicit measurement to test whether University students truly hold a neutral attitude toward negative obesity stereotypes. Considering the group specificity of PT, measuring individual tendency through a questionnaire may have weakened the empathy of participants toward the obese group and, consequently, their attitudes toward obesity stereotypes. Future research should, therefore, seek to verify the results of this study by using experimental methods to manipulate PT. Furthermore, the correlational method makes reverse causality an underlying issue. The application of experimental design will help to establish causal links between variables. Despite these limitations, this study makes valuable contributions to the literature by finding that self-other overlap and empathy both mediated the relationship between PT and positive obesity stereotypes among University students. Yet, for negative obesity stereotypes, self-other overlap emerged as the only mediator.

## Data Availability Statement

The raw data supporting the conclusions of this article will be made available by the authors, without undue reservation.

## Ethics Statement

The studies involving human participants were reviewed and approved by Inner Mongolia Normal University. Written informed consent to participate in this study was provided by the participants and underage participants' parents.

## Author Contributions

YW conceived the original idea, organized the data collection, performed the statistical analyses, and drafted the manuscript. YZ critically revised the manuscript. All the authors read and approved the final draft of the manuscript.

## Conflict of Interest

The authors declare that the research was conducted in the absence of any commercial or financial relationships that could be construed as a potential conflict of interest.

## Publisher's Note

All claims expressed in this article are solely those of the authors and do not necessarily represent those of their affiliated organizations, or those of the publisher, the editors and the reviewers. Any product that may be evaluated in this article, or claim that may be made by its manufacturer, is not guaranteed or endorsed by the publisher.

## References

[B1] Aron A. Aron E. N. Smollan D. (1992). Inclusion of other in the self scale and the structure of interpersonal closeness. J. Pers. Soc. Psychol. 63, 596–612. 10.1037/0022-3514.63.4.596

[B2] Batson C. D. Chang J. Orr R. Rowland J. (2002). Empathy, attitudes, and action: can feeling for a member of a stigmatized group motivate one to help the group. Pers. Soc. Psychol Bull. 28, 1656–1666. 10.1177/014616702237647

[B3] Batson C. D. Polycarpou M. P. Harmon-Jones E. Imhoff H. J. Mitchener E. C. Bednar L. L.. (1997). Empathy and attitudes: can feeling for a member of a stigmatized group improve feelings toward the group? J. Pers. Soc. Psychol. 72, 105–118. 10.1037/0022-3514.72.1.105 9008376

[B4] Bian N. (2015). The effect of perspective taking on the college students' elderly stereotype. [master's thesis]. Wuhan, Hubei: Central China Normal University.

[B5] Burmeister J. M. Kiefner A. E. Carels R. A. Musher-Eizenman D. R. (2013). Weight bias in graduate school admissions. Obesity 21, 918–920. 10.1002/oby.20171 23784894

[B6] Cheng L. (2017). A study on the influence of obese body shape stereotype to employment intention. [master's thesis]. Hohhot, Inner Mongolia: Inner Mongolia Normal University.

[B7] Cheng L. Zhang Y. Z. (2017). Research on the influence of perspective taking on implicit obesity stereotype. J. Inner Mongo. Norm. Univ. 30, 73–77. 10.3969/j.issn.1671-0916.2017.02.012

[B8] Corrigan P. W. David R. Amy G. Robert L. Philip R. Kyle U. W.. (2002). Challenging two mental illness stigmas: personal responsibility and dangerousness. Schizophr. Bull. 28, 293–309. 10.1093/oxfordjournals.schbul.a006939 12693435

[B9] Corrigan P. W. O'Shaughnessy J. R. (2007). Changing mental illness stigma as it exists in the real world. Aust. Psychol. 42, 90–97. 10.1080/00050060701280573

[B10] Corrigan P. W. River L. Lundin R. K. Penn D. L. Uphoff-Wasowski K. Campion J.. (2001). Three strategies for changing attributions about severe mental illness. Schizophr. Bull. 27, 187–195. 10.1093/oxfordjournals.schbul.a006865 11354586

[B11] Daníelsdóttir S. O'Brien K. S. Ciao A. (2010). Anti-fat prejudice reduction: a review of published studies. Obes. Facts 3, 47–58. 10.1159/000277067 20215795 PMC6452150

[B12] Davis M. H. (1980). A multidimensional approach to individual differences in empathy. JSAS Cat. Sel. Doc. Psychol. 10:85.

[B13] Davis M. H. Conklin L. Smith A. Luce C. (1996). Effect of perspective taking on the cognitive representation of persons: a merging of self and other. J. Pers. Soc. Psychol. 70, 713–726. 10.1037/0022-3514.70.4.713 8636894

[B14] Fowler-Brown A. G. Ngo L. H. Phillips R. S. Wee C. C. (2010). Adolescent obesity and future college degree attainment. Obesity 18, 1235–1241. 10.1038/oby.2009.463 20035280 PMC3607297

[B15] Gaertner S. L. Dovidio J. F. Anastasio P. A. Bachman B. A. (1993). The common ingroup identity model: Recategorization and the reduction of intergroup bias. Eur. Rev. Soc. Psychol. 4, 1–26. 10.1080/14792779343000004

[B16] Galinsky A. D. (2002). Creating and reducing intergroup conflict: the role of perspective-taking in affecting out-group evaluations, in Toward Phenomenology of Groups and Group Membership, ed. Sondak H. (New York, NY: Elsevier), 85–113. 10.1016/S1534-0856(02)04005-7

[B17] Galinsky A. D. Ku G. (2004). The effects of perspective-taking on prejudice: The moderating role of self-evaluation. Pers. Soc. Psychol. Bull. 30, 594–604. 10.1177/0146167203262802 15107159

[B18] Galinsky A. D. Moskowitz G. B. (2000). Perspective-taking: decreasing stereotype expression, stereotype accessibility, and in-group favoritism. J. Pers. Soc. Psychol. 78, 708–724. 10.1037/0022-3514.78.4.708 10794375

[B19] Gapinski K. D. Schwartz M. B. Brownell K. D. (2006). Can television change anti-fat attitudes and behavior? J. Appl. Biobehav. Res. 11, 1–28. 10.1111/j.1751-9861.2006.tb00017.x

[B20] Gloor J. L. Puhl R. M. (2016). Empathy and perspective-taking: examination and comparison of strategies to reduce weight stigma. Stigma Health 1, 269–279. 10.1037/sah0000030

[B21] Hair J. F. Black W. C. Babin B. J. Anderson R. E. (2010). Multivariate Data Analysis, 7th Edn. New York, NY: Pearson Prentice Hall.

[B22] Huang Q. Peng W. Simmons J. V. (2020). Assessing the evidence of perspective taking on stereotyping and negative evaluations: a p-curve analysis. Group Process. Intergroup Relat. 10.1177/1368430220957081. [Epub ahead of print].

[B23] Ku G. Wang C. S. Galinsky A. D. (2010). Perception through a perspective-taking lens: differential effects on judgment and behavior. J. Exp. Soc. Psychol. 46, 792–798. 10.1016/j.jesp.2010.04.001

[B24] Lau R. S. Cheung G. W. (2012). Estimating and comparing specific mediation effects in complex latent variable models. Organ. Res. Methods 15, 3–16. 10.1177/1094428110391673

[B25] Laurent S. M. Myers M. W. (2011). I know you're me, but who am I? Perspective taking and seeing the other in the self. J. Exp. Soc. Psychol. 47, 1316–1319. 10.1016/j.jesp.2011.05.018

[B26] Li P. F. (2017). A study of the impact ofPT on the college students' HIV/AIDS Prejudice. [master's thesis]. (Chengdu, Sichuan): Sichuan Normal University.

[B27] Myers M. W. Hodges S. D. (2012). The structure of self-other overlap and its relationship to perspective taking. Pers. Relat. 19, 663–679. 10.1111/j.1475-6811.2011.01382.x

[B28] Pettigrew T. F. Tropp L. R. (2006). A meta-analytic test of intergroup contact theory. J. Pers. Soc. Psychol. 90, 751–783. 10.1037/0022-3514.90.5.751 16737372

[B29] Podsakoff P. M. MacKenzie S. B. Lee J. Y. Podsakoff N. P. (2003). Common method biases in behavioral research: a critical review of the literature and recommended remedies. J. Appl. Psychol. 88, 879–903. 10.1037/0021-9010.88.5.879 14516251

[B30] Puhl R. M. Andreyeva T. Brownell K. D. (2008). Perceptions of weight discrimination: prevalence and comparison to race and gender discrimination in America. Int. J. Obes. 32, 992–1000. 10.1038/ijo.2008.22 18317471

[B31] Puhl R. M. Heuer C. A. (2009). The stigma of obesity: a review and update. Obesity 17, 941–964. 10.1038/oby.2008.636 19165161

[B32] Puhl R. M. King K. M. (2013). Weight discrimination and bullying. Best Pract. Res. Clin. Endocrinol. Metab. 27, 117–127. 10.1016/j.beem.2012.12.002 23731874

[B33] Puhl R. M. Latner J. D. (2007). Stigma, obesity, and the health of the nation's children. Psychol. Bull. 133, 557–580. 10.1037/0033-2909.133.4.557 17592956

[B34] Sarlio-Lähteenkorva S. (2001). Weight loss and quality of life among obese people. Soc. Indic. Res. 54, 329–354. 10.1023/A:1010939602686

[B35] Shih M. Wang E. Trahan Bucher A. Stotzer R. (2009). Perspective taking: reducing prejudice towards general outgroups and specific individuals. Group Process. Intergroup Relat. 12, 565–577. 10.1177/1368430209337463

[B36] Skorinko J. L. Sinclair S. A. (2013). Perspective taking can increase stereotyping: the role of apparent stereotype confirmation. J. Exp. Soc. Psychol. 49, 10–18. 10.1016/j.jesp.2012.07.009

[B37] Sun S. Zuo B. Wu Y. Wen F. (2016). Does perspective taking increase or decrease stereotyping? The role of need for cognitive closure. Pers. Individ. Differ. 94, 21–25. 10.1016/j.paid.2016.01.001

[B38] Taylor S. E. Brown J. (1988). Illusion and well-being: a social psychological perspective on mental health. Psychol. Bull. 103, 193–210. 10.1037/0033-2909.103.2.193 3283814

[B39] Teachman B. A. Gapinski K. D. Brownell K. D. Rawlins M. Jeyaram S. (2003). Demonstrations of implicit anti-fat bias: the impact of providing causal information and evoking empathy. Health Psychol. 22, 68–78. 10.1037/0278-6133.22.1.68 12558204

[B40] Tiggemann M. Anesbury T. (2000). Negative stereotyping of obesity in children: the role of controllability beliefs. J. Appl. Soc. Psychol. 30, 1977–1993. 10.1111/j.1559-1816.2000.tb02477.x 10751373

[B41] Tiggemann M. Rothblum E. D. (1988). Gender difference in social consequences of perceived overweight in the United States and Australia. Sex Roles 18, 75–86. 10.1007/BF00288018

[B42] Todd A. R. Bodenhausen G. V. Galinsky A. D. (2012). Perspective taking combats the denial of intergroup discrimination. J. Exp. Soc. Psychol. 48, 738–745. 10.1016/j.jesp.2011.12.011

[B43] Todd A. R. Bodenhausen G. V. Richeson J. A. Galinsky A. D. (2011). Perspective taking combats automatic expressions of racial bias. J. Pers. Soc. Psychol. 100, 1027–1042. 10.1037/a0022308 21381852

[B44] Todd A. R. Burgmer P. (2013). Perspective taking and automatic intergroup evaluation change: testing an associative self-anchoring account. J. Pers. Soc. Psychol. 104, 786–802. 10.1037/a0031999 23527849

[B45] Todd A. R. Galinsky A. D. (2014). Perspective-taking as a strategy for improving intergroup relations: evidence, mechanisms, and qualifications. Soc. Pers. Psychol. Compass 8, 374–387. 10.1111/spc3.12116

[B46] Vartanian L. R. Trewartha T. Vanman E. J. (2015). Disgust predicts prejudice and discrimination toward individuals with obesity. J. Appl. Soc. Psychol. 46, 369–375. 10.1111/jasp.12370

[B47] Vescio T. K. Sechrist G. B. Paolucci M. P. (2003). Perspective taking and prejudice reduction: the mediational role of empathy arousal and situational attributions. Eur. J. Soc. Psychol. 33, 455–472. 10.1002/ejsp.163

[B48] Wang C. S. Ku G. Tai K. Galinsky A. D. (2014). Stupid doctors and smart construction workers:PT reduces stereotyping of both negative and positive targets. Soc. Psychol. Pers. Sci. 5, 430–436. 10.1177/1948550613504968

[B49] Wang C. S. Lee M. Ku G. Leung A. K. (2018). The cultural boundaries of Perspective-taking: when and why Perspective-taking reduces stereotyping. Pers. Soc. Psychol. Bull. 44, 928–943. 10.1177/0146167218757453 29486634

[B50] Wang L. L. (2018). A study on the influence of perspective taking on homosexual stigma among the college students. [master's thesis]. [Chengdu, Sichuan]: Sichuan Normal University.

[B51] Wang Y. A. Rhemtulla M. (2021). Power analysis for parameter estimation in structural equation modeling: a discussion and tutorial. Adv. Methods Pract. Psychol. Sci. 4, 1–17. 10.31234/osf.io/pj67b

[B52] Williams L. J. Vandenberg R. J. Edwards J. R. (2009). 12 structural equation modeling in management research: a guide for improved analysis. Acad. Manag. Ann. 3, 543–604. 10.5465/19416520903065683

[B53] Zhan Z. Y. (1986). The relationship between grade, sex-role, human relationship orientation and empathy. [master's thesis]. [Taipei, Taiwan]: National Chengchi University.

[B54] Zhang F. F. Dong Y. Wang K. Zhan Z. Y. Xie L. F. (2010). Reliability and validity of the Chinese version of the interpersonal reactivity index-C. Chi. J. Clin. Psychol. 18, 25–27. 10.16128/j.cnki.1005-3611.2010.02.019

