# Supplementary material for: The Impact of Perspective Taking on Obesity Stereotypes: The Dual Mediating Effects of Self-Other Overlap and Empathy
Source: Front Psychol. 2021 Aug 12;12:643708. doi: 10.3389/fpsyg.2021.643708 (PMC8387714; doi:10.3389/fpsyg.2021.643708)
Supplement: Supplementary file 1 [file Table_1.DOCX]

Are the following items appropriate to describe you? In other words, how well does the description of each item fit you? Please circle the number that best describes you from 0 (“does not describe me well”) to 4 (“describes me very well”).

**Perspective Taking Scale**

1. I try to look at everybody’s side of a disagreement before I make a decision.

0 1 2 3 4

1. I sometimes try to understand my friends better by imagining how things look from their perspective.

0 1 2 3 4

1. I believe that there are two sides to every question and try to look at them both.

0 1 2 3 4

1. When I'm upset at someone, I usually try to “put myself in his shoes” for a while.

0 1 2 3 4

1. Before criticizing somebody, I try to imagine how I would feel if I were in their place.

0 1 2 3 4

**Empathy Scale**

1. I often have tender, concerned feelings for people less fortunate than me.

0 1 2 3 4

1. Sometimes I don't feel sorry for other people when they are having problems.

0 1 2 3 4

1. When I see someone being taken advantage of, I feel kind of protective toward them.

0 1 2 3 4

1. Other people’s misfortunes do not usually disturb me a great deal.

0 1 2 3 4

1. When I see someone being treated unfairly, I sometimes don’t feel very much pity for

Them.

0 1 2 3 4

1. I would describe myself as a pretty soft-hearted person.

0 1 2 3 4

**Inclusion of Others in Self Scale**

Please select the picture below which best describes the relationship between you and obese people.

1

2

3

4

5

6

7

Self

Obese people

**Obesity Stereotypes Questionnaire**

Although not all group members are exactly alike, group members tend to be similar on many traits and you should provide your personal opinions about the general characteristics of obese people. Do you agree with the following description of the characteristics of obese people? Please select the number from 1 (strongly disagree) to 5 (strongly agree).

1. lazy 1 2 3 4 5
2. sloppy 1 2 3 4 5
3. self-indulgent 1 2 3 4 5
4. lacking in self-discipline 1 2 3 4 5
5. clumsy 1 2 3 4 5
6. kind 1 2 3 4 5
7. warm 1 2 3 4 5
8. optimistic 1 2 3 4 5
9. simple and honest 1 2 3 4 5
10. generous 1 2 3 4 5
